# Cerebrospinal Fluid Total Tau is Increased in Normal Pressure Hydrocephalus Patients who Undergo Successful Lumbar Drain Trials

**DOI:** 10.7759/cureus.1265

**Published:** 2017-05-22

**Authors:** Geoffrey Baird, Thomas J Montine, Jason J Chang, Shu-Ching Hu, Anthony M Avellino

**Affiliations:** 1 Department of Neurological Surgery, University of Washington, Seattle, WA; 2 Pathology, Stanford University; 3 Neurology, University of Washington, Seattle, WA; 4 Neurosurgery and Pediatrics, University of Illinois College of Medicine At Peoria

**Keywords:** cerebrospinal fluid, normal pressure hydrocephalus, tau protein, lumbar drain

## Abstract

**Background:**

Idiopathic normal pressure hydrocephalus (INPH) is a neurologic disease that affects <1% of those aged >65 years, but is difficult to distinguish from other diseases that present in this age group, such as Alzheimer’s disease. Large volume lumbar puncture and an external lumbar drain trial (ELD) are used to make a clinical diagnosis of INPH, but the accuracy of ELD is suspected.

**Objective:**

To investigate proteomic cerebrospinal fluid (CSF) biomarker patterns in patients with INPH undergoing ELD to develop a quantitative diagnostic.

**Methods:**

Twenty patients with suspected INPH underwent an ELD trial and the CSF biomarkers AB_1-42_, total tau, and tau phosphorylated at amino acid 181 (p-tau) were quantified with immunoassays in specimens taken prior to ELD placement, after the ELD trial, and from ventricular samples collected at the time of permanent ventriculoperitoneal shunt placement.

**Results:**

CSF total tau was elevated, on average, in pre- and post-ELD samples from patients who failed to improve clinically during the ELD trial, but the findings were marginally significant after correction for multiple comparisons. AB_1-42 _and p-tau concentrations were not significantly different in patients who either did or did not clinically improve after the ELD.

**Conclusions:**

CSF total tau is a potential novel biomarker for suspected INPH patients who will clinically improve, or have clinically improved, after an ELD trial. The small sample size of this study, which was due to the relative rarity of this condition, indicates that larger studies are needed to confirm the utility of this approach.

## Introduction

Idiopathic normal pressure hydrocephalus (INPH) is a neurological disease that inflicts disability in approximately 0.4% of the population older than 65 years of age [1]. With the trend of aging of the society, the incidence of INPH is expected to be on the rise. It can be difficult to distinguish INPH from neurodegenerative disorders, particularly Alzheimer’s disease (AD). Due to similar manifestations between INPH and AD, and lack of specific radiographic or biochemical markers, INPH is frequently mistaken for AD, or vice versa. Manifestations of INPH, such as gait abnormality, dementia, and urinary dysfunction, are not disease-specific and they overlap with neurodegenerative diseases. Structural or functional imaging modalities such as magnetic resonance imaging and radionuclide cisternography are instrumental in differentiating INPH from neurodegenerative diseases, but currently their sensitivity and specificity are limited. Large-volume lumbar puncture and indwelling external lumbar drain (ELD) are of value in predicting surgical outcome, although their accuracy in diagnosing INPH is not conclusive. A more accurate diagnosis of INPH will need an integration of clinical data from different sources. Protein biomarkers in cerebrospinal fluid (CSF), the only body fluid in direct contact with the brain, may provide crucial biochemical information that physicians currently lack.

In this study, we measured the concentrations of several protein biomarkers in CSF in suspected INPH patients undergoing a lumbar drain trial, using samples at the time the drain was placed and at the time the drain was removed. In addition, for patients that had a positive lumbar drain trial, a ventricular sample of CSF was obtained when the patient underwent placement of a ventriculoperitoneal (VP) shunt. We hypothesized that one or more of these potential biomarkers at one or more time points would associate with the success or lack of success of the ELD trial, thus affording a potential early diagnostic modality for determining which patients might later benefit from a VP shunt.

## Materials and methods

The patient population, management protocol, and evaluation summary sections were adapted from Nakatsu, et al. [2] and are summarized below.

### Patient population

The 20 patients for the current study were drawn from the clinical neurosurgical practice of the senior author (Anthony M. Avellino), and these patients consented to have their clinical data used for research (University of Washington (UW) Institutional Review Board No. 40068, title: CSF analysis in patients with aging and neurodegenerative disorders including Normal Pressure Hydrocephalus on May 31, 2011). INPH was diagnosed by confirming that the patients had no prior history of any inciting events, including intracerebral hemorrhage, intracerebral infection such as meningitis, severe head trauma, major stroke or brain surgeries for intracerebral structural lesions such as tumors [3], and had gait problems, cognitive complaints, and/or urinary incontinence (see Table 1). The standard clinical NPH protocol involves the placement of an ELD to identify NPH patients who are likely to respond favorably to VP shunt, and candidates underwent an outpatient comprehensive neuropsychological assessment, and completed a gait evaluation with a physical therapist prior to undergoing an ELD screening trial. A lumbar drain trial was considered successful based on any objective improvement in (1) the physical therapist’s assessment, and/or (2) neuropsychological assessment. It was recommended that patients with a successful ELD trial undergo VP shunt.

**Table 1:** Demographic and clinical data from the 20 patients enrolled in this trial. ELD: External lumbar drain; VPS ICP: Initial intracranial pressure when ventriculoperitoneal shunt was placed.

Gender (M, F)	Age (years) at LD	Lumbar Drain Pressure	VPS ICP	Clinical Follow-up After VPS
F	71	17 cm H2O	10 cm H2O	Gait, Cognition, Urinary Incontinence Improved
F	72	19 cm H2O	11 cm H2O	Gait Improved, Cognition Worse
F	80	10 cm H2O	15 cm H2O	Gait, Cognition, Urinary Incontinence Improved
F	73	37 cm H2O	15 cm H2O	Gait, Cognition, Urinary Incontinence Improved
F	71	23 cm H2O	17 cm H2O	Gait, Cognition, Urinary Incontinence Improved
F	78	13 cm H2O	22 cm H2O	Gait, Cognition Improved
F	76	12 cm H2O	N/A	Failed ELD Trial/No Shunt Placed
F	78	13 cm H2O	N/A	Failed ELD Trial/No Shunt Placed
M	75	16 cm H2O	12 cm H2O	Gait Improved
M	68	13 cm H2O	13 cm H2O	Gait, Cognition, Urinary Incontinence Improved
M	76	22 cm H2O	15 cm H2O	Gait, Cognition, Urinary Incontinence Improved
M	71	9 cm H2O	15 cm H2O	No Changes
M	70	17 cm H2O	18 cm H2O	Gait, Cognition Improved
M	75	16 cm H2O	19 cm H2O	Gait, Cognition Improved
M	79	14 cm H2O	23 cm H2O	Gait, Cognition Improved
M	83	17 cm H2O	25 cm H2O	Gait, Cognition Improved
M	85	12 cm H2O	N/A	Failed ELD Trial/No Shunt Placed
M	74	18 cm H2O	N/A	Failed ELD Trial/No Shunt Placed
M	75	9 cm H2O	N/A	Failed ELD Trial/No Shunt Placed
M	86	9 cm H2O	N/A	Failed ELD Trial/No Shunt Placed

### Management protocol

NPH candidates were admitted for an ELD screening trial. The lumbar drain was placed in the operating room, the CSF pressure was obtained and recorded as an “opening CSF pressure” at the time of lumbar drain placement, and 10 ml of CSF sample (A) was obtained. The patient was then monitored on the hospital ward where we drained 100 to 250 ml of CSF per day. After 72 hours of having the lumbar drain, gait/balance evaluations and neuropsychological examinations were performed as described below, and another 10 ml of CSF sample (B) was collected. The patients, who had a successful ELD trial, were readmitted typically within 1-2 months later for VP shunt. Upon placement of the ventricular catheter for VP shunt placement, 10 ml of CSF sample (C) was obtained. The patients were then seen in the neurosurgery clinic at 1 month, 3 months, and 6 months after VP shunt.

### Evaluation summary

Gait assessment occurred prior to lumbar drain placement on the same day by a physical therapist who tested duration of standing on one foot (30 seconds maximum), the functional reach test, and timed up & go test. The baseline cognitive examination consisted of a comprehensive outpatient neuropsychological evaluation administered by a trained psychometrist and supervised by a board certified clinical neuropsychologist. Urinary function, including urgency, increased frequency, and incontinence, was assessed by patient and/or family report. After 72 hours, the lumbar drain was removed and repeat gait/balance testing and neuropsychological evaluations were completed prior to discharge. These data were compared with the baseline data obtained prior to ELD placement.

### CSF collection and evaluation

10 ml CSF samples A, B, and C were collected into screw-cap 15 ml tubes and placed in a cup of ice before transport to the laboratory for analysis. Within 30 minutes of receipt in the laboratory, samples were analyzed for cell count, total protein and glucose, and then centrifuged before separating into 1 ml aliquots in 1.5 ml screw cap vials and freezing at -80 C. CSF concentrations of AB_1-42_, total tau, and tau phosphorylated at amino acid 181 (p-tau) were quantified using the ALZBio3 kit according as previously published [4], and according to protocols of an international quality assurance program for measurement of these analytes in human CSF [5]. Statistical analysis was performed with GraphPad Prism 6, with comparisons of biomarker concentrations performed using Kruskal Wallis tests and Dunn’s correction for multiple comparisons. p < 0.05 was considered significant.

## Results

Clinical and demographic data for the patients in this study are summarized in Table 1.

**Figure 1:**
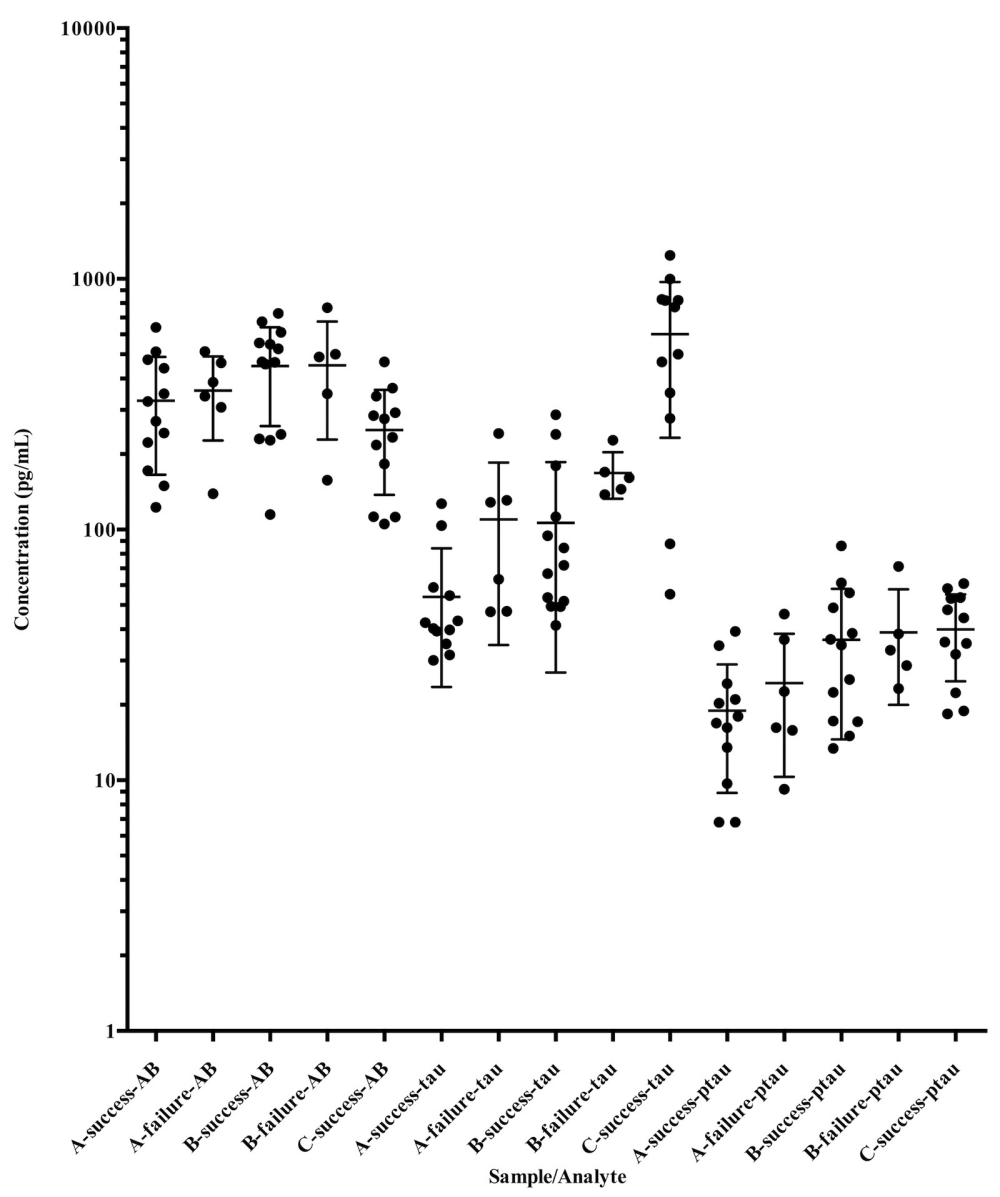
Cerebrospinal fluid (CSF) biomarker concentrations. Biomarker concentrations measured in this study are shown at different timepoints. Note that the y-axis is logarithmic.

Analyte concentrations for the A (lumbar CSF sample when lumbar drain is placed), B (lumbar CSF sample when lumbar drain is removed), and C (ventricular CSF sample taken when placing ventriculoperitoneal shunt) time point samples are shown for all patients in the study in Figure 1. For AB_1-42_ and p-tau, no comparison between samples from patients with successful or unsuccessful ELD trials was significant. For total tau, however, both the A (p = 0.056) and B (p = 0.096) time points showed marginally significantly different median analyte concentrations (after correction for making multiple comparisons) between patients with successful and unsuccessful ELD trials, with those patients having a successful ELD trial having lower average CSF total tau than those patients who had a failed ELD trial. Ventricular (C time point) CSF total tau for the successful drain trial patients was furthermore substantially elevated over lumbar CSF total tau, on average, but no comparison between patients with successful and unsuccessful drain trials could be made because patients with unsuccessful drain trials did not have a ventriculoperitoneal shunt (VPS) procedure. Neither cell counts CSF protein concentrations nor CSF glucose concentrations differed significantly between patients groups at any time points (data not shown).

## Discussion

In contrast to neurodegenerative disorders, deficits of INPH are potentially reversible if treated timely with a surgical VP shunt. Early surgical treatment of INPH, however, requires early diagnosis. If the diagnosis of INPH is delayed, irreversible disease progression ensues [6]. Therefore, early diagnosis of INPH, which entails differential diagnosis from AD, is the key to a successful outcome.

Published case series reported a range of improvement rates up to 72% in patients with INPH after shunt surgery [7-8]. At least a third of patients have no response to surgery [9], but all are equally subjected to the risks of the procedure, which include infection, over-drainage, and other postoperative complications. So far, there has been no standard algorithm for patient selection for surgery, mainly due to lack of reliable prognostic biomarkers for INPH.

Controversies abound regarding the pathogenesis of INPH. Structural changes of the ventricular wall, alteration in periventricular blood flow, free-radical reactions, and hippocampal degeneration have all been implicated [10-13]. Most studies point to reduced CSF absorption by the arachnoid granulations as the most likely culprit of INPH [14]. Pathological changes in the capillaries of the arachnoid granulations have been demonstrated in INPH [15]. Changes in expression of VEGFR-2, GFAP, TGF-ß, and other growth factors in the arachnoid granulations have also been found in INPH [16-17]. These pathological and biochemical findings have not been translated into clinical use in diagnosis or treatment of INPH.

Studies of biomarkers in body fluids have played an increasingly important role in understanding disease mechanisms and also as diagnostic tools [18]. For neurological disorders, CSF is an ideal substrate for qualitative and quantitative analysis of protein constituents of the brain. Protein biomarkers in CSF hence carry great potential for early disease diagnosis, for monitoring disease progression, and for determining treatment response. Considering the central role of CSF circulation in its pathogenesis, INPH is perhaps more likely than other similar disorders to yield informative CSF protein biomarkers. Furthermore, CSF is already obtained for pressure measurement and laboratory testing as part of standard practices when evaluating patients with possible INPH, so the approach of using CSF protein biomarkers for disease diagnosis and prognosis is especially suitable for INPH.

In this study, we found that the well-studied CSF biomarker total tau is elevated, on average, in suspected INPH patients who are destined to fail, or have failed, an ELD trial. There is some overlap between CSF total tau between these two groups and only marginal significance when assessed in the context of multiple comparisons, and so because of the small sample size of this study, it is not currently possible to assess the generalizability of these findings. While it is attractive to consider the possibility that elevated CSF in ELD non-responders is related to some pathology that increases tau release, and hence indicates that some type of damage has occurred that cannot be reversed by an ELD procedure, a larger study will be needed to confirm this finding. Furthermore, the substantially elevated CSF total tau levels identified in the ventricular CSF samples collected during the VP shunt procedure are difficult to interpret, because while they may have diagnostic or mechanistic significance, they may also simply reflect a rostrocaudal gradient of tau that could not be identified in ELD non-responders because they did not undergo VP shunt.

## Conclusions

This study was undertaken to identify potential CSF biomarkers that could aid in diagnosing INPH and predict which patients would benefit from an ELD trial or VP shunt procedure. We found that lumbar CSF total tau is on average elevated in patients who fail an ELD trial, which could indicate that a biomarker analysis could be a useful adjunct to clinical examination in diagnosing this difficult disease. Because of the rarity of INPH, however, collecting enough samples at a single site to allow for robust development and validation of a diagnostic algorithm for drain-sensitive NPH may not be possible, and hence our findings must be considered promising but preliminary. A multicenter study is warranted to investigate whether or not a diagnostic or prognostic cutoff of CSF total tau could be employed to determine which INPH patients will benefit from a shunt procedure without requiring an invasive lumbar drain trial. In addition, broader proteomic studies on the reserved samples collected in this study may identify biomarkers with more predictive or diagnostic power than the three known biomarkers studied here.

The pathophysiology responsible for the observed findings in total tau is unclear. Some evidence exists implicating the role of efflux transporters in the pathophysiology of INPH, but it is unlikely that differences in transporter activity would directly affect total tau concentrations. Conversely, as elevated CSF total tau is known to occur in several conditions with increased neuronal damage, elevated CSF total tau may indicate that a patient is suffering more severe cellular injury from INPH and is thus most sensitive to amelioration of the increased CSF pressure. Such a mechanism could potentially be studied in animal models.
